# Differential tissue expression of extracellular vesicle‐derived proteins in prostate cancer

**DOI:** 10.1002/pros.23813

**Published:** 2019-04-24

**Authors:** Diederick Duijvesz, Giovanny Rodriguez‐Blanco, A. Marije Hoogland, Esther I. Verhoef, Lennard J. Dekker, Monique J. Roobol, Geert J. L. H. van Leenders, Theo M. Luider, Guido Jenster

**Affiliations:** ^1^ Department of Urology Erasmus Medical Center Rotterdam The Netherlands; ^2^ Department of Urology Canisius Wilhelmina Hospital Nijmegen The Netherlands; ^3^ Department of Neurology Erasmus Medical Center Rotterdam The Netherlands; ^4^ Department of Pathology Erasmus Medical Center Rotterdam The Netherlands; ^5^ Department of Pathology Isala Clinics Zwolle The Netherlands

**Keywords:** biomarker, extracellular vesicles, PDCD6IP, prostate cancer, tissue microarray, XPO1

## Abstract

**Background:**

Proteomic profiling of extracellular vesicles (EVs) from prostate cancer (PCa) and normal prostate cell lines, led to the identification of new candidate PCa markers. These proteins included the nuclear exportin proteins XPO1 (also known as CRM1), the EV‐associated PDCD6IP (also known as ALIX), and the previously published fatty acid synthase FASN. In this study, we investigated differences in expression of XPO1 and PDCD6IP on well‐characterized prostate cancer cohorts using mass spectrometry and tissue microarray (TMA) immunohistochemistry to determine their diagnostic and prognostic value.

**Methods:**

Protein fractions from 67 tissue samples (n = 33 normal adjacent prostate [NAP] and n = 34 PCa) were analyzed by mass spectrometry (nano‐LC‐MS‐MS). Label‐free quantification of EVs was performed to identify differentially expressed proteins between PCa and NAP. Prognostic evaluation of the candidate markers was performed with a TMA, containing 481 radical prostatectomy samples. Samples were stained for the candidate markers and correlated with patient information and clinicopathological outcome.

**Results:**

XPO1 was higher expressed in PCa compared to NAP in the MS data analysis (*P* > 0.0001). PDCD6IP was not significantly higher expressed (*P* = 0.0501). High cytoplasmic XPO1 staining in the TMA immunohistochemistry, correlated in a multivariable model with high Gleason scores (*P* = 0.002) and PCa‐related death (*P* = 0.009).

**Conclusion:**

High expression of cytoplasmic XPO1 shows correlation with prostate cancer and has added clinical value in tissue samples. Furthermore, as an extracellular vesicles‐associated protein, it might be a novel relevant liquid biomarker.

**Abbrrviations**
CRM1: chromosomal maintenance 1
CRPC: castration resistant prostate cancer
EVs: extracellular vesicles
FASN: fatty acid synthetase N
IHC: immunohistochemistry
LC: liquid chromatography
LFQ: label free quantification
MS: mass spectrometry
NAP: normal adjacent prostate
PCa: prostate cancer
PDCD6IP: programmed cell death 6 interacting protein; also known as ALIX
PSA: prostate specific antigen
RNA: ribonucleic acid
TMA: tissue microarray
TURP: transurethral resection of the prostate
XPO1: exportin‐1

## 1 INTRODUCTION

Biomarker discovery via extracellular vesicles (EVs; often referred to as exosomes) released by (cancer) cells, has been the focus of many research groups in the last decade.^1^, ^2^ Based on their biogenesis and secretion pathway, they contain low‐abundant, cancer‐specific proteins, and RNAs that could be of interest in identifying novel biomarkers.^3^ With respect to prostate cancer (PCa), several EV‐derived candidate biomarkers have been revealed.^4^, ^5^, ^6^, ^7^, ^8^, ^9^, ^10^

Although multiple markers have been proposed as candidates for several malignancies, the majority has been identified and validated in EVs derived from cell culture. Few of the candidate biomarkers have been validated on larger groups of patient samples. Because this validation step is rarely taken, it remains difficult to elucidate the full potential of EV markers, which limits its translation and clinical implementation.^11^, ^12^

Our own efforts, by using state‐of‐the‐art mass spectrometry, has led to the discovery of some candidate markers of which XPO1 (also known as CRM1), FASN, and PDCD6IP (also known as ALIX) were found to have the highest potential.^7^ The objective of this study was to investigate whether the PCa EV‐associated expression could be reproduced in tissue analyses of larger cohorts of patients. Result for FASN has been published previously.^13^

## 2 MATERIALS AND METHODS

### 2.1 Mass spectrometry

Protein fractions from tissue RNA isolations with RNA‐Bee of 67 PCa tissue samples (33 NAP and 34 PCa) were selected and stored at −80°C as described in Rodriguez et al.^13^ Samples were thawed and 50 µL precipitated with cold acetone and microcentrifugation. After 10 minutes, the supernatant was removed and the pellet washed twice with cold acetone. The supernatant was removed and 50 µL of 0.1% RapiGest (Waters Corporation, Milford, MA) in 50 mM NH_4_HCO_3_ was added to the protein pellet. The protein pellet was dissolved by external sonification for 5 minutes at 70% amplitude at room temperature (Digital Sonifier model 450, Branson, Danbury, CT). The proteins were reduced with 10 mM dithiothreitol at 60°C for 30 minutes. After cooling down to room temperature, it was alkylated with 50 mM iodoacetamide for 30 minutes, and digested overnight with 8 µL trypsin (Promega, Madison, WI). Subsequently, 6 µL of 5% TFA was added to inactivate digestion and incubated for 30 minutes at 37°C. Samples were centrifuged at maximum speed for 60 minutes at 4°C and the supernatant was transferred to new tubes. A total of 5 µL was diluted 40 times and subsequently transferred to LC vials for LC‐MS analysis. Upon analysis, 2 µL was injected to the nano‐LC. After preconcentration and washing of the sample it was loaded on to a C18 column (PepMap C18, 75 mm ID × 500 mm, 2 μm particle, and 100 Å pore size; Thermo Fisher Scientific, Bremen, Germany) using a linear 90 minutes gradient (4%‐25% acetonitrile/H20; 0.1% formic acid) at a flow rate of 250 nL/minute. The separation of the peptides was monitored by a UV detector (absorption at 214 nm). The nano‐LC was coupled to a nanospray source of a Q‐Exactive plus mass spectrometer (Thermo Fisher Scientific, Bremen, Germany). Full scan MS spectra (m/z 400‐1600) in profile mode were acquired in the Orbitrap with a resolution of 70 000 after the accumulation of an AGC target of 1 × 10^6^. The top 12 peptide signals (charge‐state 2^+^ and higher) were isolated (1.6 Da window) and fragmented by HCD (higher‐energy collision, normalized collision energy 28.0) and measured in the Orbitrap with an AGC target of 50 000 and a resolution of 17 500. Maximum fill times were 100 ms for the full scans and 60 ms for the MS/MS scans. The dynamic exclusion was activated, after the first time a precursor was selected for fragmentation it was excluded for a period of 30 seconds using a relative mass window of 10 ppm. Lock mass correction was activated to improve mass accuracy of the survey scan.

Label‐free quantitation was performed using Progenesis LC‐MS Software (version 3.0; Nonlinear Dynamics Ltd., Newcastle‐upon‐Tyne, UK) following our previously reported methodology.^14^, ^15^ To get quantitative data, we selected only proteins identified by three or more peptides for statistical analysis of protein abundance between groups. Duplicates in identified sequences as a consequence of peak tailing were removed to avoid false positives. Technical replicates of each sample were randomly analyzed within the measurement period and no significant changes in the number of identified proteins were observed between replicates and quality control measurements.

### 2.2 Tissue microarray

A tissue microarray (TMA) was constructed as published previously.^16^ Briefly, 481 men were selected from the European Randomized Study of Screening for prostate cancer (ERSPC), who had undergone radical prostatectomy for PCa.^17^ From each patient sample, three representative cores (diameter 0.6 mm) were taken and placed in nine paraffin blocks. Patient information and clinical follow data were recorded via the ERSPC protocol and stored in a central study database.

For immunohistochemical (IHC) staining the tissues slides were mounted on aminoacetylsilane coated glass slides (Starfrost, Berlin, Germany), deparaffinized with xylene and dehydrated in ethanol. The slides were placed in 0.3% hydrogen peroxide in PBS for 20 minutes to block endogenous peroxidase activity. Microwave pretreatment was performed for 15 minutes in tris (hydroxymethyl)aminomethane‐ethylenediaminetetraacetic acid (pH 9.0). Subsequently, the slides were incubated with PDCD6IP (1:400), FASN (1:50), and XPO1 (1:50) antibodies, overnight at 4°C. The EnVision DAKO kit (DAKO, Glostrup, Denmark) was used for chromogenic visualization. Counterstaining was performed with hematoxylin, which was followed by dehydration and mounting in malinol (Chroma‐Geselschaft, Körgen, Germany).

Staining intensities of each antibody were scored independently by two investigators (DD, AMH) as negative (0; no staining), weak (1; only visible at high magnification), moderate (2; visible at low magnification), and strong (3; striking at low magnification).^18^ Based on previous IHC staining results, for XPO1 a score was assigned to both nuclear staining and cytoplasmic staining.^7^ For PDCD6IP only the cytoplasmic expression was scored. In cases of staining heterogeneity, the highest expression levels were used for statistical analysis. After scoring, the average intensity for the triplicate cores was calculated. When a core was missing or no cancer was observed, this respective case was excluded from the analysis. In a combined session consensus on expression value was reached in all cases.

Statistical association of staining intensities and clinic‐pathological features (PSA at diagnosis, Gleason score (GS), pT‐stage, surgical margins, biochemical recurrence, local recurrence, overall death, and PCa‐related death) were performed with SPSS (version 17, SPSS Inc., Chicago, IL) by using Pearson's χ^2^ tests and Student's *t* tests. A multivariable analysis was performed to determine the contribution of each individual variable. A *P*‐ < 0.05 was considered to be statistically significant.

## 3 RESULTS

### 3.1 Protein expression by mass spectrometry

**Figure 1:**
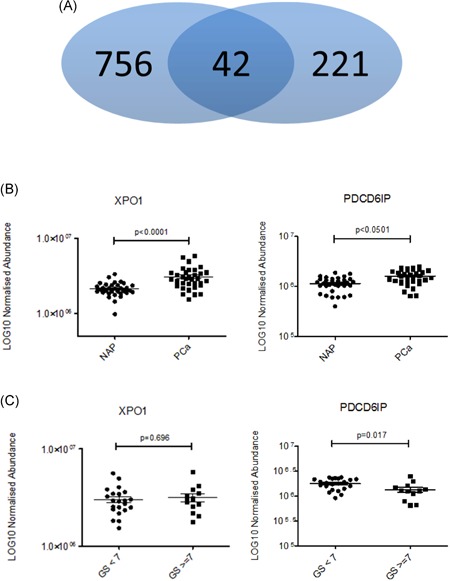
A, Overlap of proteins between the discovery set of extracellular vesicle‐associated proteins (n = 263)^7^ and the proteins differentially expressed between prostate cancer (PCa) and normal adjacent prostate (NAP) tissue (n = 798).^13^ B, Protein expression (LOG10 normalized) of XPO1 and PDCD6IP in NAP (n = 33) and PCa tissue (n = 34) and in C, Gleason score < 7 (n = 22) vs Gleason score ≥ 7 (n = 12) [Color figure can be viewed at wileyonlinelibrary.com]

We previously published a list with proteins (n = 263) that were identified in EVs from normal prostate (PNT2C2 and RWPE‐1) and PCa (VCaP and PC346c) cell lines by using mass spectrometry.^7^ From this list, 10 proteins were identified as higher expressed in PCa‐derived EVs of which expression of 3 proteins (XPO1, FASN, and PDCD6IP) were further analyzed for EV and tissue expression. For a first validation, we compared the 263 proteins to a shotgun mass spectrometry database recently published which 34 PCa (n = 22 GS 6, n = 12 GS≥ 7) and 33 NAP tissues were compared using label‐free quantification.^19^ In this database, a total of 2865 proteins were identified from which 798 proteins were statistically significant differentially expressed between normal prostate and PCa (FDR < 0.01). When compared to the list of EV‐derived proteins, 42 of these proteins showed overlap (Figure 1A and Table 1).

**Table 1:** Proteins that were significantly differentially expressed between extracellular vesicles (EVs) from prostate cancer (PCa) cells and non‐PCa cells^7^ compared to MS‐MS protein expression in PCa and NAP tissue^13^

		Accession number	Peptide count	Unique peptides	Confidence score	ANOVA (p)	Max fold change	Highest mean expression	Lowest mean expression
Higher expression in VCaP and PC346c PCa‐derived EVs									
Fatty acid synthase	FASN	P49327	103	98	147,44	0,000000	2,95	PCa	NAP
Exportin‐1	XPO1	O14980	7	7	7,92	0,000001	1,52	PCa	NAP
Polyadenylate‐binding protein 1	PABPC1	P11940	14	11	16,92	0,001511	1,43	NAP	PCa
CD9 antigen	CD9	P21926	4	4	7,98	0,050249	1,30	NAP	PCa
Programmed cell death 6‐interacting protein	PDCD6IP	Q8WUM4	30	27	35,77	0,245175	1,10	PCa	NAP
Elongation factor 1‐α 2	EEF1A2	Q05639	13	5	17,92	0,235288	2,23	PCa	NAP
Ubiquitin‐60S ribosomal protein L40	UBA52	P62987	7	1	10,96	0,309769	3,36	PCa	NAP
Basal cell adhesion molecule	BCAM	P50895	11	10	12,95	0,472126	1,23	PCa	NAP
Vacuolar protein sorting‐associated protein 28 homolog	VPS28	not identified by mass‐spectrometry in PCa tissue							
Actin‐related protein 3B	ACTR3B	not identified by mass‐spectrometry in PCa tissue							
Higher expression in PNT2C2 and RWPE‐1 normal prostate‐derived EVs									
Annexin A2(P07355)	ANXA2	P07355;A6NMY6	28	28	38,85	0,000000	1,50	NAP	PCa
UDP‐glucose 6‐dehydrogenase(O60701)	UGDH	O60701	24	22	31,86	0,000000	2,03	PCa	NAP
Ras‐related protein Rap‐1A(P62834)	RAP1A	P62834	8	1	8,92	0,000011	1,69	NAP	PCa
14‐3‐3 protein theta(P27348)	YWHAQ	P27348	20	12	24,89	0,000201	1,33	PCa	NAP
T‐complex protein 1 subunit epsilon(P48643)	CCT5	P48643	17	14	18,87	0,000301	1,43	PCa	NAP
78 kDa glucose‐regulated protein(P11021)	HSPA5	P11021	36	28	49,82	0,000304	1,47	PCa	NAP
Chloride intracellular channel protein 1(O00299)	CLIC1	O00299	13	12	17,94	0,000486	1,22	PCa	NAP
Keratin, type I cytoskeletal 9(P35527)	KRT9	P35527	4	4	3,93	0,000631	5,87	PCa	NAP
14‐3‐3 protein epsilon(P62258)	YWHAE	P62258	22	19	24,87	0,010096	1,27	PCa	NAP
Sodium/potassium‐transporting ATPase subunit β‐3(P54709)	ATP1B3	P54709	4	4	4,98	0,026991	1,16	PCa	NAP
Ras‐related protein Rab‐10(P61026)	RAB10	P61026	6	3	6,98	0,028100	1,24	PCa	NAP
Pyruvate kinase isozymes M1/M2(P14618)	PKM2	P14618	29	29	45,85	0,030393	1,10	NAP	PCa
Ras‐related protein Rab‐1A(P62820)	RAB1A	P62820	8	3	10,95	0,033276	1,19	PCa	NAP
α‐Enolase(P06733)*	ENO1	P06733	30	20	44,82	0,043375	1,22	PCa	NAP
Hemoglobin subunit β (P68871)	HBB	P68871;P69891;P69892	18	11	35,87	0,043867	1,53	NAP	PCa
Peroxiredoxin‐1(Q06830)	PRDX1	Q06830	15	11	19,92	0,083975	1,17	PCa	NAP
ADP‐ribosylation factor 1(P84077)	ARF1	P84077	10	3	11,96	0,132790	1,22	PCa	NAP
Keratin, type II cytoskeletal 2 epidermal(P35908)	KRT2	P35908	6	1	6,96	0,150597	1,24	NAP	PCa
Sodium/potassium‐transporting ATPase subunit β‐1(P05026)	ATP1B1	P05026	1	1	1,98	0,186607	1,10	NAP	PCa
Catenin β‐1(P35222)	CTNNB1	P35222	19	13	21,91	0,190862	1,14	PCa	NAP
Ras GTPase‐activating‐like protein IQGAP1(P46940)	IQGAP1	P46940	33	28	37,85	0,198229	1,12	PCa	NAP
Ras‐related C3 botulinum toxin substrate 1(P63000)	RAC1	P63000	6	2	7,97	0,216742	1,14	PCa	NAP
Phosphoglycerate kinase 1(P00558)	PGK1	P00558	28	26	42,79	0,246349	1,06	NAP	PCa
Sodium/potassium‐transporting ATPase subunit α‐1(P05023)	ATP1A1	P05023	28	25	32,83	0,346299	1,07	PCa	NAP
14‐3‐3 protein beta/alpha(P31946)	YWHAB	P31946	15	7	19,93	0,370719	1,07	PCa	NAP
Tubulin α‐1A chain(Q71U36)	TUBA1A	Q71U36;A6NHL2	25	2	37,82	0,377593	1,06	PCa	NAP
Lactadherin(Q08431)	MFGE8	Q08431	1	1	1,00	0,518547	1,37	PCa	NAP
EH domain‐containing protein 4(Q9H223)	EHD4	Q9H223	8	6	7,95	0,533687	1,02	PCa	NAP
Triosephosphate isomerase(P60174)	TPI1	P60174	17	15	30,90	0,560817	1,05	PCa	NAP
Importin subunit β‐1(Q14974)	KPNB1	Q14974	16	16	18,91	0,592615	1,04	PCa	NAP
Basigin(P35613)	BSG	P35613	3	3	2,94	0,744475	1,03	NAP	PCa
Junctional adhesion molecule A(Q9Y624)	F11R	Q9Y624	3	3	2,98	0,767732	1,07	PCa	NAP
Protein DJ‐1(Q99497)	PARK7	Q99497	16	15	21,89	0,790992	1,01	NAP	PCa
Adenosylhomocysteinase (P23526)	AHCY	P23526	20	18	23,85	0,906165	1,01	PCa	NAP
Integrin α‐6(P23229)	ITGA6	not identified by mass‐spectrometry in PCa tissue							
Actin, aortic smooth muscle(P62736)	ACTA2	not identified by mass‐spectrometry in PCa tissue							
Potassium‐transporting ATPase α chain 2(P54707)	ATP12A	not identified by mass‐spectrometry in PCa tissue							
4F2 cell‐surface antigen heavy chain(P08195)	SLC3A2	not identified by mass‐spectrometry in PCa tissue							
CD151 antigen(P48509)	CD151	not identified by mass‐spectrometry in PCa tissue							
Coxsackievirus and adenovirus receptor(P78310)	CXADR	not identified by mass‐spectrometry in PCa tissue							
Prostaglandin F2 receptor negative regulator(Q9P2B2)	PTGFRN	not identified by mass‐spectrometry in PCa tissue							
Putative heat shock protein HSP 90‐β2(Q58FF8)	HSP90AB2P	not identified by mass‐spectrometry in PCa tissue							
Putative heat shock protein HSP 90‐β‐3(Q58FF7)	HSP90AB3P	not identified by mass‐spectrometry in PCa tissue							

Abbreviation: NAP, normal adjacent prostate.

Our previously identified candidate PCa‐EV biomarkers XPO1 (*P* < 0.0001) and FASN (*P* < 0.0001) were higher expressed in PCa tissue, while PDCD6IP was borderline not significantly higher expressed (*P* = 0.0501) (Table 1; Figure 1B). Interestingly, polyadenylate‐binding protein 1 (PABCP1) was higher expressed in PCa‐derived EVs but showed significantly lower expression in PCa tissue when compared to NAP (*P* = 0.0015). Four other proteins (CD9, EEF1A2, UBA52, and BCAM) were not significantly differentially expressed in the tissue proteomics. The PCa‐derived EV proteins VPS28 and ACTR3B were not identified in the tissue validation set. Proteins that were higher expressed in normal prostate cell line EVs were also cross‐validated on MS‐MS data of tissue samples and are shown in Table 1. Although 15 of the 34 were differentially expressed in both datasets, only 4 of the 15 showed the same direction of higher expressed in EVs from immortalized normal prostate epithelial cell lines and higher expressed in NAP tissue.

When expression of proteins was compared, XPO1 was significantly higher expressed in PCa (Figure 1B), but no difference (*P* = 0.696) was observed between low risk (GS 6) and intermediate/high‐risk PCa (GS ≥ 7) (Figure 1C). Interestingly, PDCD6IP was significantly lower expressed in intermediate/high risk in prostate tissue (*P* = 0.017) (Figure 1C).

### 3.2 Protein expression by tissue microarray immunohistochemistry

For independent validation of XPO1 and PDCD6IP, immunohistochemistry (IHC) was performed on the TMA. Patient characteristics and clinicopathological parameters are shown in Table 2 and were previously published by Hoogland et al.^16^ Briefly, the mean age at the time of radical prostatectomy was 64.7 years; follow‐up was 113.3 months. Gleason score after radical prostatectomy was <7 in 265 (55.1%), 7 in 188 (39.1%), and >7 in 28 (5.8%) patients. Surgical margins were negative in 362 (75.3%) patients. Biochemical recurrence was observed in 119 (24.7%) patients after an average of 40.9 months. Staining intensities of the candidate biomarkers could not be assessed in 57 of the 481 samples (11.8%) because the tumor was absent.

**Table 2:** Patient characteristics and clinicopathological parameters of the prostate samples after treatment by radical prostatectomy (n = 481) as was published by Hoogland et al^16^

Patient characteristics and clinico‐pathological parameters	Total number of patients (%)	Mean (variation)
Age at diagnosis, y		64.5 (55‐75)
>60	n = 411 (85.4)	
>65	n = 260 (54.0)	
>70	n = 57 (10.6)	
Follow‐up, mo		113.3 (0‐204)
PSA levels at diagnosis, ng/mL		7.2 (0.3‐125)
>2.5	n = 440 (91.5)	
>4	n = 333 (69.2)	
>10	n = 62 (12.9)	
Gleason sum		
<7	n = 199 (41.4)	
=7	n = 188 (39.1)	
>7	n = 28 (5.8)	
Pathological T‐stage (TNM 2002)		
T2a	n = 84 (17.5)	
T2b	n = 10 (2.1)	
T2c	n = 246 (51.1)	
T3a	n = 93 (19.3)	
T3b	n = 17 (3.5)	
T4	n = 28 (5.8)	
Surgical margins		
Positive	n = 119 (24.7)	
Negative	n = 362 (75.3)	
Biochemical recurrence, mo		40.9 (0‐205)
Yes	n = 119 (24.7)	
No	n = 362 (75.3)	
Local recurrence, mo		110.0 (6‐146)
Yes	n = 24 (5.0)	
No	n = 457 (95.0)	
Overall death, mo		113.7 (0‐202)
Yes	n = 112 (23.3)	
No	n = 368 (76.6)	
Prostate cancer related death		
Yes	n = 12 (10.7)	
No	n = 74 (66.0)	
Unknown	n = 26 (23.2)	

Abbreviation: PSA, prostrate specific antigen.

**Figure 2:**
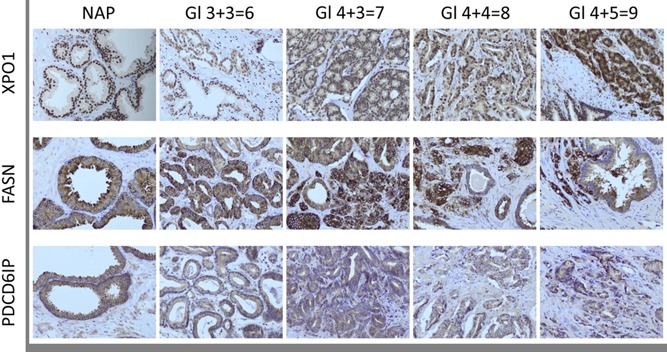
Immunohistochemical staining of XPO1, FASN, and PDCD6IP on normal adjacent prostate (NAP) and prostate cancer (PCa) with increasing Gleason scores (GS). Picture was partially published before.^7^ [Color figure can be viewed at wileyonlinelibrary.com]

Antibody IHC verification and impression of the tissue staining of the three candidate protein markers was published previously and further expanded as depicted in Figure 2.^7^ XPO1, PDCD6IP, and FASN stainings were observed in all samples, mainly in epithelial cells. We noticed that the XPO1 expression varied within and between the nuclear and cytoplasmic compartments. Nuclear XPO1 expression was present in 98.4% of cases and cytoplasmic expression was observed in 74.5% of cases. XPO1 showed strong nuclear and low cytoplasmic expression in luminal cells in NAP.^7^ With the progression of PCa and increasing GS, cytoplasmic XPO1 expression increased (Figure 2). PDCD6IP showed high expression in both luminal and basal cells in NAP.

Subsequently, we evaluated the association between protein expression intensities of our three candidate biomarkers and PSA at diagnosis, GS, pT‐stage, surgical margins, biochemical recurrence, local recurrence, overall death and PCa‐related death (Tables 3, 4; Table S1). Nuclear XPO1 expression and PDCD6IP did not correlate with any clinicopathological parameter. High cytoplasmic XPO1 expression correlated with GS ≥ 7 (*P* = 0.002) and PCa‐specific death after multivariate analysis (*P* = 0.009) (Table 4). All other parameters were not significantly different. The FASN TMA analyses have recently been published as part of the tissue proteomics study and revealed that expression among the PCa samples was higher in GS < 7 and GS = 7 (49.0% and 34.6%, respectively) than in GS > 7 (5.4%). This is in agreement with previous studies.^6^, ^19^

**Table 3:** Scored staining intensity and distribution of the cytoplasmic and nuclear XPO1 in our tissue microarray

Expression nuclear XPO1	0	1	2	3	Total	*P*‐value
PSA at diagnosis						
≤10 ng/mL	5	66	217	119	396	0.012
>10 ng/mL	1	16	35	8	60	
Total	6	71	252	127	456	
Gleason score						
<7	5	40	129	74	248	0.145
7	0	26	105	50	181	
>7	1	5	19	3	28	
Total	6	71	253	127	457	
pT‐stage						
pT2	5	49	177	93	324	0.345
pT3a/b	1	14	65	26	106	
pT4	0	8	11	8	27	
Total	6	71	253	127	457	
PSA at diagnosis						
≤10 ng/mL	104	236	55	1	396	0.920
>10 ng/mL	15	38	7	0	60	
Total	119	274	62	1	456	
Gleason score						
<7	79	146	23	0	248	0.002
7	40	109	31	1	181	
>7	1	19	8	0	28	
Total	120	274	62	1	457	
pT‐stage						
pT2	92	195	36	1	324	0.221
pT3a/b	23	61	22	0	106	
pT4	5	18	4	0	27	
Total	120	274	62	1	457	

Intensity was scored as negative (0; no staining), weak (1; only visible at high magnification), moderate (2; visible at low magnification), or strong (3; striking at low magnification). Staining intensities were correlated with patient characteristics after radical prostatectomy

Abbreviation: PSA, prostrate specific antigen.

**Table 4:** Univariate and multivariate correlation of clinicopathological parameters and staining intensities of PDCD6IP, nuclear XPO1, and cytoplasmic XPO1. A *P* < 0.05 was considered as statistically significant

	PSA	Gleason score	pT stage	Biochemical recurrence				Local recurrence				Death				Prostate specific death			
				Univariate		Multivariate		Univariate		Multivariate		Univariate		Multivariate		Univariate		Multivariate	
	*P*‐value	*P*‐value	*P*‐value	HR	*P*‐value	HR	*P*‐value	HR	*P*‐value	HR	*P*‐value	HR	*P*‐value	HR	*P*‐value	HR	*P*‐value	HR	*P*‐value
Nuclear XPO1	0.012	0.145	0.345	0.97 (0.75‐1.26)	0.831	0.97 (0.75‐1.26)	0.831	0.59 (0.32‐1.06)	0.079	0.59 (0.31‐1.12)	0.109	1.08 (0.83‐1.41)	0.575	1.10 (0.83‐1.46)	0.520	0.96 (0.42‐2.17)	0.913	1.70 (0.56‐5.17)	0.351
Cytoplasmic XPO	0.920	0.002	0.221	1.04 (0.77‐1.41)	0.783	1.04 (0.77‐1.41)	0.783	1.30 (0.65‐2.62)	0.462	1.03 (0.49‐2.12)	0.946	1.14 (0.84‐1.54)	0.413	1.11 (0.80‐1.53)	0.546	1.90 (0.92‐3.19)	0.084	3.03 (1.33‐6.93)	0.009
PDCD6IP	0.490	0.581	0.439	1.16 (0.83‐1.64)	0.386	1.16 (0.83‐1.64)	0.386	1.22 (0.54‐2.79)	0.631	1.51 (0.65‐3.54)	0.340	1.04 (0.74‐1.46)	0.825	1.05 (0.75‐1.49)	0.765	1.05 (0.39‐2.81)	0.926	1.50 (0.39‐5.74)	0.558

Abbreviation: HR, hazards ratio; PSA, prostrate specific antigen.

## 4 DISCUSSION

In this study we investigated whether previously identified PCa EV‐derived proteins were differentially expressed in PCa tissue from patients using mass spectrometry and immunohistochemistry. We found that XPO1 was associated with PCa in mass spectrometry and with higher GS using IHC on our TMA. PDCD6IP was not associated with adverse clinicopathological characteristics.

XPO1 (also known as CRM1) mediates nuclear export of proteins and RNAs and its differential expression has been linked to multiple types of cancer.^20^, ^21^, ^22^ These transported proteins play a role in tumor signaling pathways, including the AR‐pathway.^23^, ^24^ XPO1 has already been identified as a marker for several malignancies.^21^ We identified this protein to be higher expressed in EVs derived from the VCaP PCa cell line.^7^ In this current study, we observed higher expression in PCa tissue as compared to NAP, which could explain the increased EV expression. From the TMA, we noticed that when GS increased, nuclear staining decreased and cytoplasmic XPO1 location increased. This provides us with an additional explanation for the increased presence of this 123 kDa nuclear export protein in EVs: a shift towards cytoplasmic expression could increase the random chance or even active escorting of XPO1 into extracellular vesicles.

When correlated to clinicopathological parameters, we observed a significant correlation between higher XPO1 cytoplasmic expression with higher GS (7 or higher) and disease‐specific death. Therefore, this finding implies that there seems to be a clinical role as a tissue marker regarding prognosis for PCa. The correlation with disease‐specific death could only be addressed in 12 patients. Because of the limited number of patients with PCa‐related death, we should be careful to draw conclusions regarding the correlation of cytoplasmic XPO1 and this clinical parameter.

Interestingly, recent reports have been published on the functional role of XPO1 and the effect of cancer by inhibition of XPO1‐mediated transport. Administration of selective inhibitors of nuclear transport (SINE) such as Selinexor, have led to an enrichment of tumor suppressor proteins in the nucleus.^25^ This subsequently resulted in apoptosis, reduction of tumor spreading and improved overall survival in preclinical models in PCa.^26^, ^27^, ^28^ Clinical studies are being performed to reveal the real potential of XPO1‐directed therapy.

FASN has already been described as a potential marker for PCa.^29^, ^30^ A recent study by Hamada et al^31^, showed that expression of this protein on biopsies could be a marker for the upgrading of GS after radical prostatectomy. Furthermore, Wu et al^32^ showed that this protein is useful for the diagnosis of PCa. However, both studies were performed on relatively small groups (<100 patient samples). Although we previously showed higher expression of FASN in PCa EVs, Rodriguez‐Blanco et al^19^, could only observe a statistically significant difference with normal prostate tissue in our TMA. When expression was compared with clinicopathological parameters, no statistically significant difference was observed. So far, FASN could be used as a marker for the diagnosis PCa, but the expression in normal cells is also relatively high. This makes it difficult for distinguishing between disease and healthy tissue.

PDCD6IP has scarcely been reported as a tumor marker or as having a role in tumor biology. PDCD6IP (also referred to as ALIX) is involved in endocytosis, multivesicular body biogenesis, apoptosis, membrane repair, and directly related to EV formation.^33^, ^34^ Although PDCD6IP is used as a general marker for EVs, the transformation of healthy cells to cancerous cells could interfere with EV formation and therefore the presence of PDCD6IP could be altered. Our current study showed no statistical difference of expression between NAP and PCa tissue, nor did we find decreased PDCD6IP expression in patients with GS≥7 in our TMA. Furthermore, PDCD6IP did not correlate to any clinicopathological parameters. We conclude that over‐representation of PDCD6IP in EVs from VCaP and PC346c is not explained by a general higher expression of this protein in PCa.

Although PDCD6IP differential expression in EVs could not be validated with tissue MS‐MS and TMA IHC, it would be interesting to see whether this marker still shows clinical potential when an easily applicable EV‐specific assay (such as an ELISA) is applied.

From our mass spectrometry and TMA analyses, we have learned that overexpression and cytoplasmic compartmentalization provides an explanation for the increased presence of XPO1 in PCa‐derived EVs. The absence of a correlation between tissue and EV expression for PCDC6IP suggest that also other mechanisms play a role in EV‐mediated secretion. The most obvious would be specific escorting of proteins in extracellular vesicles via the ESCRT system.^35^, ^36^ Whether this pathway is changed in cancer development and progression is not fully known.^37^

Besides XPO1, FASN, and PDCD6IP, our analyses also provided more data for new candidate EV biomarkers: The 16 additional proteins that were identified in EVs and also differentially expressed in the tissue MS analyses. It is worthwhile to investigate whether these PCa tissue‐dysregulated, EV‐detectable proteins are EV biomarkers in other cell lines and in clinical samples. From this list, PABPC1, CLIC1, RAB10, and PKM2 have been identified as a potential marker for (prostate) cancer. 38, 39, 40, 41 High expression of ANXA2 has been shown to have an unfavorable prognosis in multiple malignancies.^42^ Within this group of potential biomarkers, it is of interest to note that the level of expression in EVs from normal versus PCa cell lines is not always in the same direction as the NAP versus PCa tissue MS (Table 1). Besides the comparison of a few 2D grown cell lines with patient tissue samples, changes in cytoplasmic subcellular location and specific EV sorting of proteins can explain such apparent discrepancies.

XPO1, FASN, and PCDC6IP are known or expected to be intra‐vesicular proteins and a detection assay to determine their levels in EVs from bodyfluids will likely involve multiple steps: EV isolation and disruption followed by protein measurement using enzyme‐linked immunosorbent assays (ELISA), related immune‐assays or mass spectrometry.^3^, ^43^ Robust and reproducible EV isolation and protein assays are still under development and until these technologies are standardized, large scale intra‐vesicular protein biomarker validation and clinical implementation are difficult to realize. This is different for EV membrane‐associated proteins that can be detected with antibodies while the vesicle remains intact. Standard ELISA‐like assays might capture and detect the EV protein of interest, directly from biofluids.^12^, ^43^

## 5 CONCLUSIONS

In this study, we investigated previously‐identified EV‐derived markers on large cohorts of patient tissue samples for validation of diagnostic and prognostic differential expression. High expression of cytoplasmic XPO1 shows a strong correlation with PCa progression, while no differential tissue expression of PDCD6IP was observed. The increase in cytoplasmic XPO1 during the progression of PCa can explain the higher abundance in secreted EVs.

## Supporting information

Supplementary information

Supplementary information
