# Supplementary material for: Differential tissue expression of extracellular vesicle‐derived proteins in prostate cancer
Source: Prostate. 2019 Apr 24;79(9):1032–42. doi: 10.1002/pros.23813 (PMC6594141; doi:10.1002/pros.23813)
Supplement: Supplementary file 1 — Supplementary information [file PROS-79-1032-s001.docx]

| **Expression PDCD6IP** |  | **1** | **2** | **3** | **Total** | ***p*-value** |
| --- | --- | --- | --- | --- | --- | --- |
| PSA at diagnosis  ≤10 ng/ml  >10 ng/ml  Total |  | 7  2  9 | 204  33  237 | 189  24  213 | 400  59  459* | 0,490 |
| Gleason score  <7  7  >7  Total |  | 5  4  0  9 | 123  98  16  237 | 126  76  12  214 | 254  178  28  460* | 0,581 |
| pT-stage  pT2  pT3a/b  pT4  Total |  | 6  3  0  9 | 162  60  41  104 | 161  41  12  214 | 319  104  27  460* | 0,439 |
