# Supplementary material for: Differential tissue expression of extracellular vesicle‐derived proteins in prostate cancer
Source: Prostate. 2019 Apr 24;79(9):1032–42. doi: 10.1002/pros.23813 (PMC6594141; doi:10.1002/pros.23813)
Supplement: Supplementary file 2 — Supplementary information [file PROS-79-1032-s002.docx]

**Suppl. Table 1:** Scored staining intensity and distribution of the PDCD6IP. Intensity was scored as negative (0; no staining), weak (1; only visible at high magnification), moderate (2; visible at low magnification), or strong (3; striking at low magnification). Staining intensities were correlated with patient characteristics after radical prostatectomy.
